# Exploring the familial role of social responsiveness differences between savant and non-savant children with autism

**DOI:** 10.1038/s41598-020-59209-7

**Published:** 2020-02-10

**Authors:** Edan Daniel, Idan Menashe

**Affiliations:** 1 Department of Cognitive and Brain Sciences, Ben-Gurion University of the Negev, Beer Sheva, Israel; 2 Zlotowski Center for Neuroscience, Ben-Gurion University of the Negev, Beer Sheva, Israel; 3 Department of Public Health, Ben-Gurion University of the Negev, Beer Sheva, Israel

**Keywords:** Human behaviour, Comorbidities

## Abstract

Savant syndrome is a phenomenon whereby individuals with cognitive impairments have one or more outstanding abilities, inconsistent with their general intellectual functioning. Approximately 50% of savant individuals have autism spectrum disorder (ASD), and 10–30% of people with ASD have savant skills. To shed additional light on this considerable overlap, we compared autistic traits as measured by the Social-Responsiveness-Scale (SRS) between 712 children with at least one reported savant skill, as determined by designated questions from the ADI-R questionnaire (savant group), and 2,032 non-savant children from the Simons-Simplex-Collection (SSC) database. We also examined SRS scores of the parents of these children and compared parent-child differences in SRS scores between the savant and non-savant groups. Savant children had significantly lower SRS scores (less deficiencies) compared to non-savant children (P < 0.05), while no such differences were observed among their parents. Further intra-familial analyses revealed weak pairwise-correlations (r = −0.015–0.141) between SRS scores of parents and their children, and significantly larger parent-child differences in standardized SRS scores within savant families (P < 0.05). These findings suggest that the less severe autistic traits among savant children with ASD compared to other people with ASD is not likely to be a familial trait.

## Introduction

Savant syndrome is a fascinating phenomenon whereby individuals with cognitive impairments have one or more outstanding abilities, inconsistent with their general intellectual functioning^1^. The special skills typically occur in five areas - music, art, calendar calculating, mathematics or mechanical/visual-spatial skills, and are usually associated with outstanding memory that is restricted to the area of expertise^2–5^. In most cases, the condition is evident from early childhood^5–7^, however few cases of acquired savant skills have been documented following a CNS injury or disease^8,9^.

Interestingly, there is a high concordance between savant syndrome and autism spectrum disorder (ASD) where approximately 50% of savant individuals have also ASD^5,10,11^, and 10–30% of persons with ASD have exceptional abilities^4,12,13^. Another similarity between these two conditions is that both have a similar male-to-female ratio of 4-6:1^5,14^. In the general population, a relation between special abilities and ASD-like-traits has been observed, where children with special abilities show elevated ASD-like traits compared to those without such abilities^15–18^. In contrast, within ASD populations, Individuals with special abilities tend to have less ASD-like traits, or – elevated social skills^13,19^.

The considerable concordance between ASD and savant syndrome could stem from a shared etiology of these two conditions, and/or from a mechanism by which one condition predisposes the emergence of the other. Theories supporting the first option include the “Enhanced Perceptual Functioning” model that suggests that the over-functioning of brain regions involved in perceptual functions among people with ASD may be also responsible for the high prevalence of special abilities in these people^20,21^. Veridical mapping has been further proposed as the specific mechanism that accounts for the role of perception in the manifestation of exceptional skills among people with ASD^22–24^. A few rare genetic syndromes support these theories where people with these syndromes have both autistic features and specific enhanced skills^25–27^. Alternatively, the “Empathizing–Systemizing” theory, suggests that the attention to detail observed in ASD individuals occurs in the service of achieving an ultimate understanding of a system, and may predispose the emergence of savant skills^28^. Similarly, in their “Fractionable Triad Proposal”, Happé and Vital showed that the greater number of ASD-like traits found in savant-autistic children was particularly due to increased restricted and repetitive behaviors and interests (RRBIs), while their social and communication impairments were somewhat reduced^17^. Therefore, they suggested that the autistic characteristic of a detail focused cognitive style (weak coherence) predisposes to talent, and that the presence of savant skills may aid social competence. Other studies found no indication that elevated RRBIs predict the existence of savant skills, although they did find that autistic savants have better cognition and social skills than people with ASD without any exceptional talent^4,13,29^.

Today, the high concordance between the savant syndrome and ASD is still an enigma. The main goal of this study was to examine whether children with ASD and exceptional skills (i.e. autistic savants) have different autistic characteristics, than other children with ASD, and whether such differences are familial or not. To address this question, we compared various autistic traits as measured by the Social Responsiveness Scale (SRS) questionnaire^30^ between children with ASD and their parents. These analyses allowed us to assess whether the elevated social skills observed among individuals with ASD and savant skills may have a familial origin.

## Data and Methods

### Data source

The data for this study was derived from the Simons Simplex Collection (SSC; version 15)^31^ which includes a wide variety of clinical and genetic information from 2,852 families in which only one child has a diagnosis of ASD (simplex families). Presence of savant skills was determined using five designated questions in the Autism Diagnostic Interview-Revised (ADI-R) questionnaire^32^, as defined in previous, large scale studies^4,12,13,29,33^. A subject was defined as currently having an exceptional skill that is “definitely above the subject’s general level of ability and above the general population’s normal level” if he/she had a score of 2 (“isolated skill, not used functionally/meaningfully”) or 7 (“isolated skill, used meaningfully”) in at least one of the following special skill items: Visuospatial ability (item 88); musical ability (item 90); drawing skill (item 91); reading ability (item 92); and computational ability (item 93).

Next, we used data from the Social Responsiveness Scale (SRS) questionnaire^30^ filled by the parents of children with ASD to identify the presence and extent of autistic traits in this sample. We also used data from the adult version of the SRS questionnaire that was filled by both child’s parents on each other, to assess the parental social responsiveness in an equivalent manner. Each item in the SRS questionnaire is rated on a scale of 0 (not true) to 3 (almost always true), and the total score serves as an index for the severity of autistic traits where a higher total score indicates a more severe social deficit. We used these raw SRS scores (of both child and adult versions) throughout this study. Further, the SRS questionnaire can be divided into five subscales according to the domain of autistic trait (social awareness, social communication, social cognition, autistic mannerisms and social motivation), and each of these subscales can be assessed separately.

### Statistical analysis

First, we compared demographic features and primary ASD diagnosis measures from the ADOS and ADI-R tools using chi-square (for nominal variables) and Mann-Whitney U tests (for continuous variables). We further used independent samples t-tests or Mann-Whitney U tests (where a violation of the normal distribution assumption occurred) to compare the total SRS scores and scores of the different SRS domains between the savant and non-savant groups, as well as between specific savant subtypes, and the non-savant group. Significant differences in SRS scores were reevaluated in multivariate logistic regression models that controlled for the children’s sex and age.

Finally, we aimed at evaluating the familial contribution to the child’s autistic characteristics. Hence, we examined the correlations between SRS scores of children and their parents. In addition, we computed SRS z-scores for children (standardized by the other children SRS scores) and their parents (standardized by the same gender parent SRS scores) to bring the SRS scores of all family members to the same scale. Then, we applied a two-way ANOVA of repeated measures to these SRS z-scores followed by paired t-tests to assess the effects of the parent autistic characteristics and the child’s savant status as well as their interaction on the child’s SRS score. Statistically significant results were determined at the P < 0.05 level, with a false-discovery rate (FDR) correction for multiple testing^34^ when necessary.

### Compliance with ethical standards

All procedures and experimental protocols performed in this study involving human participants were approved by the institutional research committee at Ben-Gurion University. The study was performed in accordance with the guidelines and regulations of the 1964 Helsinki declaration and its later amendments or comparable ethical standards. Informed consent was obtained from a parent and/or legal guardian of all participants in this study.

## Results

Of the 2,852 children with autism at the SSC database, 2,744 (96.2%) had data from both SRS and ADI-R questionnaires and hence were included in our study. Of these, 712 children (25.9%) had at least one reported exceptional talent (see methods) and were consequently included in the savant group in this study. There were no statistically significant differences in gender nor race between the savant and non-savant groups. However, savant children were, on average, six months older than non-savant children at the time of ADOS administration (112.9 ± 42.7 vs. 106.7 ± 42.7 respectively; Table 1). In addition, savant children had significantly higher IQ, nonverbal communication, and social abilities compared to non-savant children (Table 1). Notably, these differences, as well as all the findings that are presented in further analyses did not change after accounting for the age difference between the groups using regression models (Supplementary Table S1).

**Table 1:** Core descriptive variables of children from the SSC database.

Variable N (%); Mean (SD)		Savant 711 (25.9%)	Non-Savant 2029 (74.1%)	Effect size	P-value
Gender (Male)		628 (88.3%)	1741 (85.8%)	1.26^b^	0.104^d^
Race	White	570 (80.5%)	1582 (78.7%)	1.00^b^	0.467^d^
	African American	25 (3.5%)	85 (4.2%)	0.82^b^	
	Asian	30 (4.2%)	81 (4.0%)	1.03^b^	
	Other	26 (3.7%)	105 (5.2%)	0.69^b^	
	More-than-one-race	57 (8.1%)	156 (7.8%)	1.01^b^	
Age in months^**a**^	Mean (SD)	112.77 (42.61)	106.50 (42.59)	0.15^c^	<0.001^e^
	Range	48–214	48–216		
IQ	Total	95.79 (24.65)	75.88 (27.18)	0.75^c^	<0.001^e^
	Verbal	91.59 (28.14)	73.12 (30.87)	0.61^c^	<0.001^e^
	Nonverbal	98.23 (22.47)	79.60 (25.62)	0.75^c^	<0.001^e^
ADI-R	Verbal Communication	16.34 (4.20)	16.56 (4.29)	−0.05^c^	0.249^e^
	Nonverbal Communication	8.77 (3.46)	9.43 (3.42)	−0.19^c^	<0.001^e^
	Restricted & Repetitive Behaviors	6.82 (2.61)	6.42 (2.45)	0.16^c^	<0.001^e^
	Social interactions	19.83 (5.62)	20.50 (5.74)	−0.12^c^	0.007^e^
ADOS	Social Communication	12.78 (4.06)	13.54 (4.18)	−1.83^c^	<0.001^e^
	css	7.43 (1.68)	7.45 (1.68)	−0.01^c^	0.838 ^e^
	Restricted & Repetitive Behaviors	3.71 (2.01)	4.07 (2.07)	−0.17^c^	<0.001^e^
	Social Affect	10.56 (3.86)	11.38 (4.03)	−0.21^c^	<0.001^e^
SRS	Total	95.96 (26.42)	98.75 (27.2)	−0.10^c^	0.018^e^
	Awareness	12.29 (3.50)	12.66 (3.72)	−0.10^c^	0.021^e^
	Cognition	19.90 (5.59)	18.78 (5.63)	−0.16^c^	<0.001^e^
	Communication	32.78 (9.82)	33.69 (9.60)	−0.09^c^	0.036^e^
	Mannerism	18.34 (6.65)	18.80 (6.90)	−0.07^c^	0.126^e^
	Motivation	14.65 (5.69)	14.83 (5.77)	−0.03^c^	0.474^e^

^a^Age at the time of ADOS administration. It is the same age at which both ADI-R and SRS data were obtained for these children. ^b^Odds ratio ^c^Cohen’s d. ^d^Chi-square ^e^Mann-Whitney-U test.

**Figure 1:**
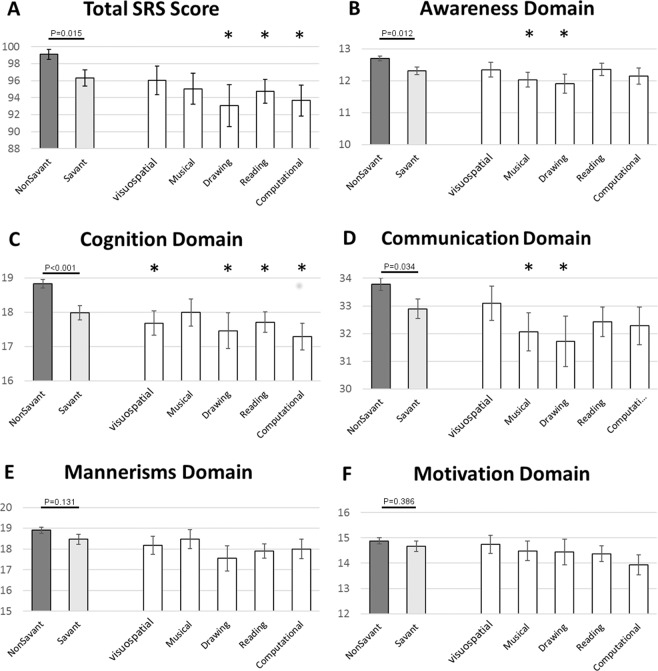
Comparison of raw SRS scores between the savant and non-savant groups. Mean ± SE of SRS scores are displayed for children in the non-savant group (Dark Gray bars), children with any savant skill (Light Gray bars), as well as for children with specific savant skills (White bars). Asterisks indicate statistically significant difference between groups of specific savant skills and non-savants at (P_FDR_ < 0.05). (**A**) Total SRS scores; (**B**) awareness domain scores; (**C**) cognition domain scores; (**D**) communication domain scores; (**E**) mannerisms domain scores; (**F**) motivation domain scores.

To study differences in social responsiveness between savant and non-savant children with ASD, we compared the SRS scores between these two groups (Fig. 1, gray columns, and Supplementary Fig. S1). There were significant differences between these two groups in the total SRS score (*P* = *0.015*, Cohen’s d = 0.103), as well as in the scores of the awareness domain (*P* = *0.012*, Cohen’s d = 0.106), the cognition domain (*P* < *0.001*, Cohen’s d = 0.151), and in the communication domain (*P* = *0.034*, Cohen’s d = 0.090) with savants having on average lower scores (less deficiencies) than non-savants in all of these domains. There were non-significant differences between the savant and non-savant groups in the mannerism and motivation domain scores. We further examined the SRS scores of children with specific reported talents (Fig. 1, white columns). In general, SRS scores of all the different savant subgroups were lower than the scores of the non-savants (less deficiencies). Specifically, statistically significant differences after correcting for multiple testing included lower total SRS score in the computational, reading, and drawing subgroups (*P*_*FDR*_ = *0.04*, Cohen’s d = 0.200; *P*_*FDR*_ = *0.001*, Cohen’s d = 0.161; and *P*_*FDR*_ = *0.04*, Cohen’s d = 0.220; respectively), lower awareness scores in the drawing and musical subgroups (*P*_*FDR*_ = *0.04*, Cohen’s d = 0.214; P_FDR_ = 0.04, Cohen’s d = 0.180; respectively), lower cognition scores in the computational, reading, drawing, and visuospatial subgroups (*P*_*FDR*_ = *0.001*, Cohen’s d = 0.273; *P*_*FDR*_ = *0.001*, Cohen’s d = 0.200; *P*_*FDR*_ = *0.04*, Cohen’s d = 0.242; and *P*_*FDR*_ = *0.03*, Cohen’s d = 0.205; respectively), and lower communication scores in the drawing and musical subgroups (*P*_*FDR*_ = *0.04*, Cohen’s d = 0.207; *P*_*FDR*_ = *0.04*, Cohen’s d = 0.172; respectively).

**Figure 2:**
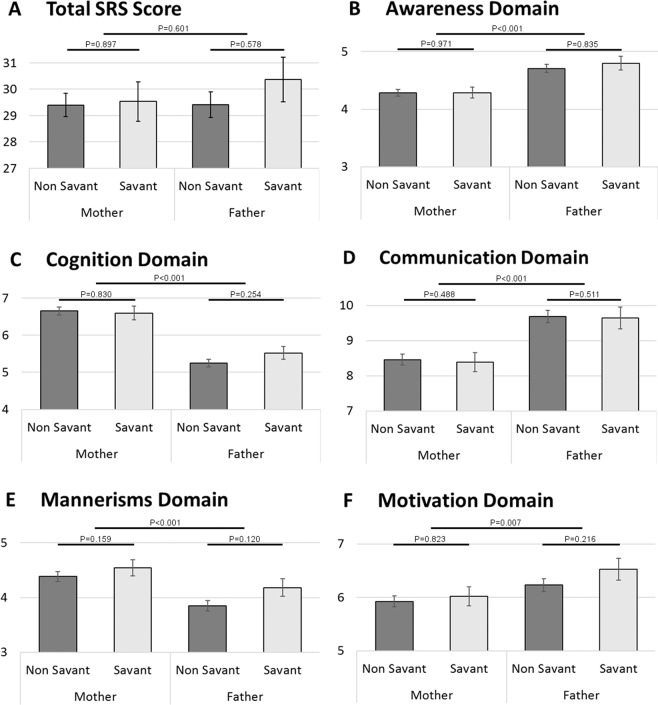
Comparison of raw SRS scores between parents of children with ASD. Mean ± SE of SRS scores are displayed for parents of children in the non-savant group (Dark Gray bars), and in the savant group (Light Gray bars). Statistically significant differences between fathers and mothers of children with ASD are seen across all SRS domains but not in the total SRS score. (**A**) Total SRS scores; (**B**) awareness domain scores; (**C**) cognition domain scores; (**D**) communication domain scores; (**E**) mannerism domain scores; (**F**) motivation domain scores.

Next, we examined the adult SRS scores of both mothers and fathers of children in this study. No significant differences in autistic traits were seen between parents of the savant and non-savant groups (P > 0.06, Cohen’s d < 0.012; Fig. 2). However, significant gender differences (P < 0.05, Cohen’s d > 0.050) were observed in all five domains of the SRS questionnaire where fathers performed better (lower SRS scores) in the cognition and mannerisms domains, whereas mothers had better awareness, communication and motivation skills. These differences were consistent among parents of both savant and non-savant children with ASD except in the motivation domain where the gender difference in the savant group was only marginally significant (*P* = *0.057, Cohen’s d* = *0.069*). Interestingly, no such gender differences in SRS scores were seen among the children of these parents (Supplementary Fig. S2).

**Figure 3:**
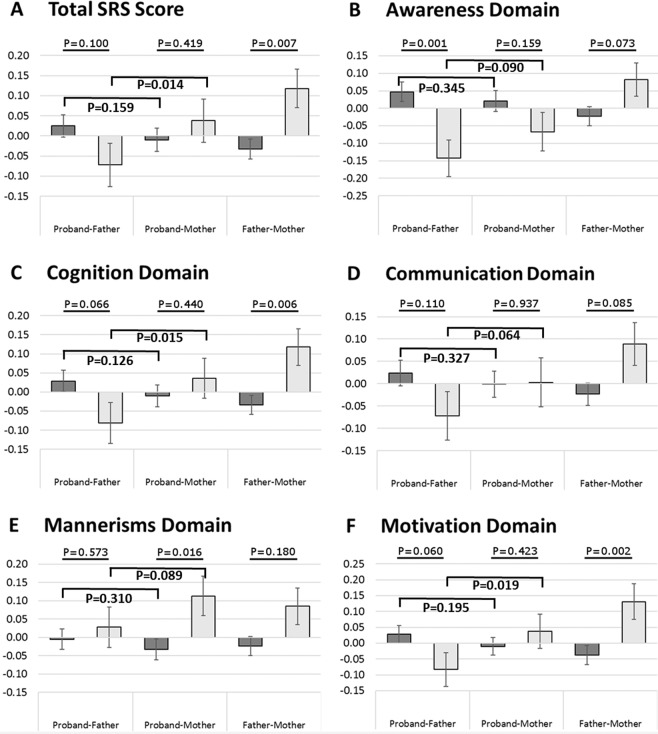
Parent-child pairwise differences of standardized SRS scores. The mean difference in standardized SRS score between children and their fathers, children and their mothers, and between fathers and mothers of these children are depicted for the non-savant group (Dark Gray bars) and savant group (Light Gray bars). (**A**) Total SRS scores; (**B**) awareness domain scores; (**C**) cognition domain scores; (**D**) communication domain scores; (**E**) mannerism domain scores; (**F**) motivation domain scores.

Finally, we examined the relationships between SRS scores of each child and their parent(s). First, we evaluated the parent-child pairwise correlations in SRS scores. Overall, parents had SRS scores that were more similar to each other than to their children across all SRS subscales and in both savant and non-savant groups (supplementary Table S2). Next, we applied a repeated measures ANOVA to these data followed by paired t-test analyses (See Methods) to explicitly scrutinize the possible reasons for the differences in SRS scores between child-parent pairs, which is not possible when comparing group means. The results of this analysis highlight three notable trends (Fig. 3): (1) children with autism were more similar to their mothers than to their fathers (smaller pairwise differences, with the only exception in the autistic-mannerisms domain); (2) there were larger pairwise differences in the SRS scores among family trios of the savant groups than among the non-savant families; and (3) pairwise differences of the savant and non-savant groups were consistently in different directions (i.e. positive differences for the savant pairs and negative differences in the non-savant pairs or vice versa). These trends resulted in a significant parent (mother/father) X savant status (savant/non-savant) interaction (*P* < *0.05*) in the total SRS score and all its subdomains except the communication domain (*P* = *0.11*) suggesting that parent-child differences in SRS scores are remarkably larger in families of autistic savants. The child–father and child–mother differences in raw SRS scores are depicted in supplementary Table S3.

## Discussion

The main goal of this study was to examine whether children with ASD and exceptional skills (i.e. autistic savants) have different autistic characteristics, as measured by the SRS, than other children with ASD, and whether such differences are familial or not. We show that parents of children with and without savant skills do not have different social responsiveness characteristics. Yet, in line with previous studies^13,19^, children with autism and savant skills have significantly lower SRS scores (less deficiencies) in a range of social impairment domains, as well as having higher IQ, and lower scores in the ADOS and ADI-R tests than non-savant autistic children. The observed differences in age and IQ between these two groups could contribute to the reported differences is social skills. Alternatively, the less apparent autistic features among the savant children with ASD could lead to a delayed diagnosis in these children. Of note, these differences tend to be larger among children with specific skill types (Fig. 1, white columns), suggesting that individuals with different savant skills may have distinct patterns of autistic traits.

Our findings of no significant difference in the autistic mannerisms domain of the SRS between the savant and non-savant groups join other findings (e.g.^4,29^) that partially negate the “Fractionable Triad Proposal” which suggests that the autistic characteristic of a detail focused cognitive style (weak coherence, RRBIs) predisposes to talent^17,21,35,36^. In contrast, the observation that the savant group is characterized by better social awareness in our study is compatible with the suggestion that the presence of special skills may aid social competence^17,37^. Importantly, similar results were seen when we compared each of the five savant subgroups (defined by their talents) to the non-savant group. This may also support the premise that the presence of exceptional talent among people with ASD enhances their social skills, regardless of the type of talent.

Interestingly, there were significant gender differences in all domains of the adult version of the SRS questionnaire, however no such differences were seen in the child questionnaires. A possible explanation of such observation is a rater bias, due to the different gender ratio of the raters of these questionnaires; Child questionnaires are more commonly filled by mothers than by fathers^38^ whereas adult questionnaires are always filled by one parent, on the second parent. Thus, the observed differences in SRS scores between the mothers and fathers in this study most likely reflect differences in the rater’s judgment in the different rater groups. Yet, the possibility that these differences reflect true differences in social behavior is still valid. Unfortunately, the lack of information regarding the gender of the child SRS rater in our dataset disallowed us from testing this hypothesis.

Finally, we observed larger intra-familial differences in standardized SRS scores among the savant families than in the non-savant families. This finding implies that the differences in autistic traits between the savant and non-savant children with ASD are unlikely driven by familial factors. We therefore suggest that the relationship between talent and social behavior observed in this study and others^13,17^ is more likely a result of one trait nurturing the other. Nevertheless, our data cannot indicate whether the existence of exceptional talent improves social competence, or whether the predisposition for better social skills promotes talent. In addition, a mutual synergism between these two traits whereby the existence of one enhances the other and vice-versa is also a valid possibility. Further empirical studies of talented children with ASD are thus warranted to gain better understanding of the mechanisms underlying talent in these children. Results of such studies will have remarkable social and clinical implications to people with ASD as well as to other human beings.

The strengths of this study include its large sample size, especially the relatively large number of female subjects which is a recurring limitation among studies of autistic populations. The large sample size allowed us to examine gender-based differences, which was not possible in previous, smaller samples. Nevertheless, our findings should be interpreted in the context of several limitations. First, the savant definition was based on parent report, and was not validated by empirical tests. Using the questions from the ADI-R to determine savant skills in children with ASD may result in either underestimation or overestimation of savant skills in the study. Multiple other large-scale studies (e.g.^4,12,13,29,33^ used the parental reports to determine exceptional talents among children with ASD. Some of these studies, which used both parental reports and empirical tests of some of the reported skills^4,39^, suggested that parental reports provide a good proxy for their child’s exceptional talent. Nevertheless, this approach is certainly imperfect, and may cause inclusion of empirically non-talented children in the savant group. Second, despite the relatively large sample size of this study, the classification of the savant group into talent-specific subgroups resulted in small sample sizes, which together with the need of multiple testing correction reduced the statistical power to draw robust conclusions about differences between these groups. Third, the Simons simplex sample is not a genuine representation of the autistic population in the US or elsewhere. Thus, caution should be taken in generalization of the conclusions of this study. Fourth, this is a cross-sectional study whereby single measurements of both the SRS and savant skills were taken at the same time. Therefore, we could not determine which of these traits predispose the other one. Finally, we did not have data regarding the occurrence of exceptional talents among the parents of the children in our dataset and hence could not assess whether the talents reported in the children are inherited or acquired.

## Conclusions

Our results suggest that the better social responsiveness observed among people with ASD and savant skills compared to other people with ASD is not likely to be a familial trait. Further studies are required to examine whether any of these distinct autistic features predisposes the other, or whether they co-develop simultaneously.

## Supplementary information

Supplementary Tables and Figures.
