# Supplementary material for: Exploring the familial role of social responsiveness differences between savant and non-savant children with autism
Source: Sci Rep. 2020 Feb 10;10:2255. doi: 10.1038/s41598-020-59209-7 (PMC7010762; doi:10.1038/s41598-020-59209-7)
Supplement: Supplementary file 1 — Supplementary Tables and Figures. [file 41598_2020_59209_MOESM1_ESM.docx]

Exploring the familial role of social responsiveness differences between savant and non-savant children with autism.

Authors: Edan Daniel^1, 2^, Idan Menashe^2,3^

1. Department of Cognitive and Brain Sciences, Ben-Gurion University of the Negev, Beer Sheva, Israel
2. Zlotowski Center for Neuroscience, Ben-Gurion University of the Negev, Beer Sheva, Israel
3. Public Health Department, Ben-Gurion University of the Negev, Beer Sheva, Israel

Corresponding author:

Idan Menashe, PhD

Department of Public Health

Faculty of Health Sciences

Ben-Gurion University of the Negev

Beer-Sheva, Israel

Email: [idanmen@bgu.ac.il](mailto:idanmen@bgu.ac.il)

Tel: +972-8-6477456; Fax: +972-8-6477638

**Supplementary Table S1: A logistic regression model for differences between savant and non-savant children with ASD adjusted for age and sex.**

|  | B | OR* | 95%CI (OR) | P-value |
| --- | --- | --- | --- | --- |
| Age at ADOS | 0.004 | 1.004 | 1.002 - 1.007 | <0.001 |
| Sex (male) | 0.203 | 1.225 | 0.924 - 1.565 | 0.130 |
| SRS total | -0.004 | 0.996 | 0.992 – 0.998 | 0.01 |

* Odds ratio (OR)

**Supplementary Table S2: Pearson’s correlations between SRS scores of ASD family members**

| **Family pair** | **SRS subscale** | **Pearson’s r (ALL)** | **Pearson’s r (Savant)** | **Pearson’s r (non-Savant)** |
| --- | --- | --- | --- | --- |
| **Child-Mother** | Awareness | 0.118^***^ | 0.116^**^ | 0.119^***^ |
|  | Cognition | 0.104^***^ | 0.114^**^ | 0.104^***^ |
|  | Communication | 0.098^***^ | 0.073 | 0.107^***^ |
|  | Mannerisms | 0.134^***^ | 0.118^**^ | 0.142^***^ |
|  | Motivation | 0.113^***^ | 0.141^***^ | 0.104^***^ |
|  | Total | 0.126^***^ | 0.120^**^ | 0.130^***^ |
| **Child-Father** | Awareness | 0.034 | -0.015 | 0.05^*^ |
|  | Cognition | 0.104^***^ | 0.085^*^ | 0.111^***^ |
|  | Communication | 0.076^***^ | 0.032 | 0.092^***^ |
|  | Mannerisms | 0.083^***^ | 0.047 | 0.096^***^ |
|  | Motivation | 0.107^***^ | 0.055 | 0.126^***^ |
|  | Total | 0.103^***^ | 0.069 | 0.116^***^ |
| **Father-Mother** | Awareness | 0.229^***^ | 0.247^***^ | 0.222^***^ |
|  | Cognition | 0.295^***^ | 0.299^***^ | 0.294^***^ |
|  | Communication | 0.290^***^ | 0.288^***^ | 0.291^***^ |
|  | Mannerisms | 0.260^***^ | 0.271^***^ | 0.255^***^ |
|  | Motivation | 0.035 | 0.028 | 0.037 |
|  | Total | 0.307^***^ | 0.307^***^ | 0.307^***^ |

* p < 0.05, ** p < 0.01, *** p < 0.001

**Supplementary Table S3: Child-parent differences in raw SRS scores.**

| SRS domain | Child-Father  (Mean difference ± SE) | | Child-Mother  (Mean difference ± SE) | |
| --- | --- | --- | --- | --- |
|  | **savant** | **Non-savant** | **savant** | **Non-savant** |
| Total | 66.08±1.21 | 69.34±0.72 | 65.49±1.25 | 69.22±0.73 |
| Awareness | 7.79±0.17 | 8.18±0.10 | 7.26±0.17 | 7.74±0.10 |
| Cognition | 11.74±0.27 | 12.61±0.16 | 12.87±0.26 | 14.02±0.15 |
| Communication | 24.26±0.45 | 25.50±0.26 | 23.10±0.47 | 23.95±0.27 |
| Mannerisms | 13.77±0.29 | 14.41±0.17 | 14.12±0.29 | 14.94±0.17 |
| Motivation | 8.52±0.27 | 8.89±0.16 | 8.12±0.28 | 8.58±0.17 |

**Figure S1**

**
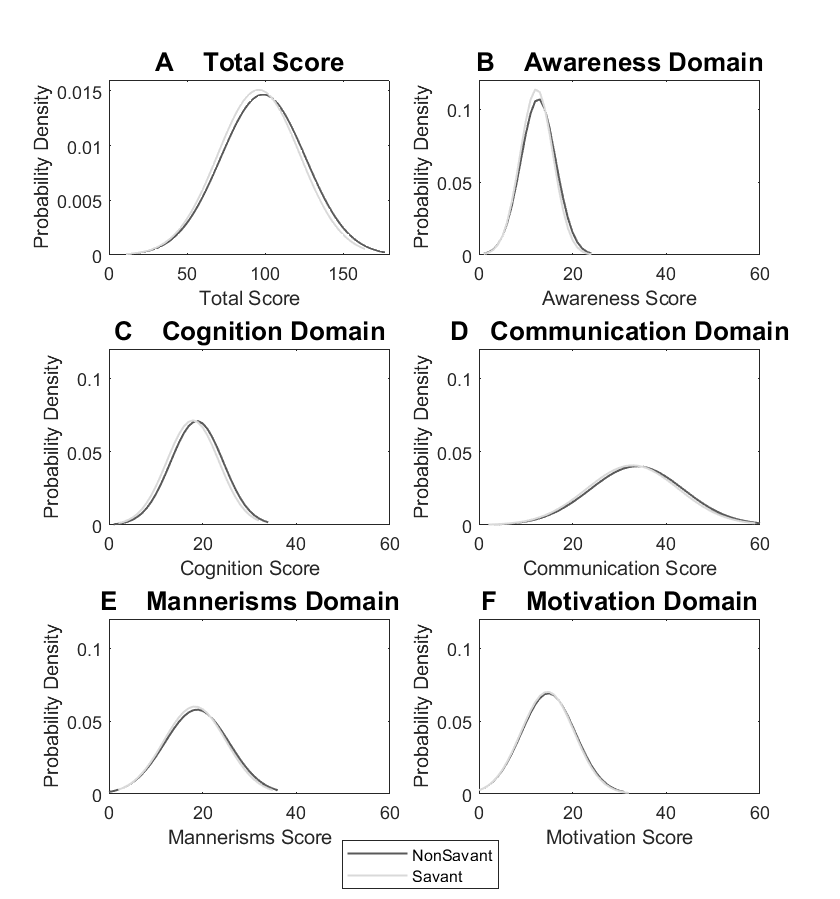
**

**Figure S1**

Density plots of raw SRS scores. Smoothed density plots of the SRS scores in the non-savant (Dark Gray bars), and savant (Light Gray bars) groups. (A) Total SRS scores; (B) awareness domain scores; (C) cognition domain scores; (D) communication domain scores; (E) mannerism domain scores; (F) motivation domain scores.

**Figure S2**

**
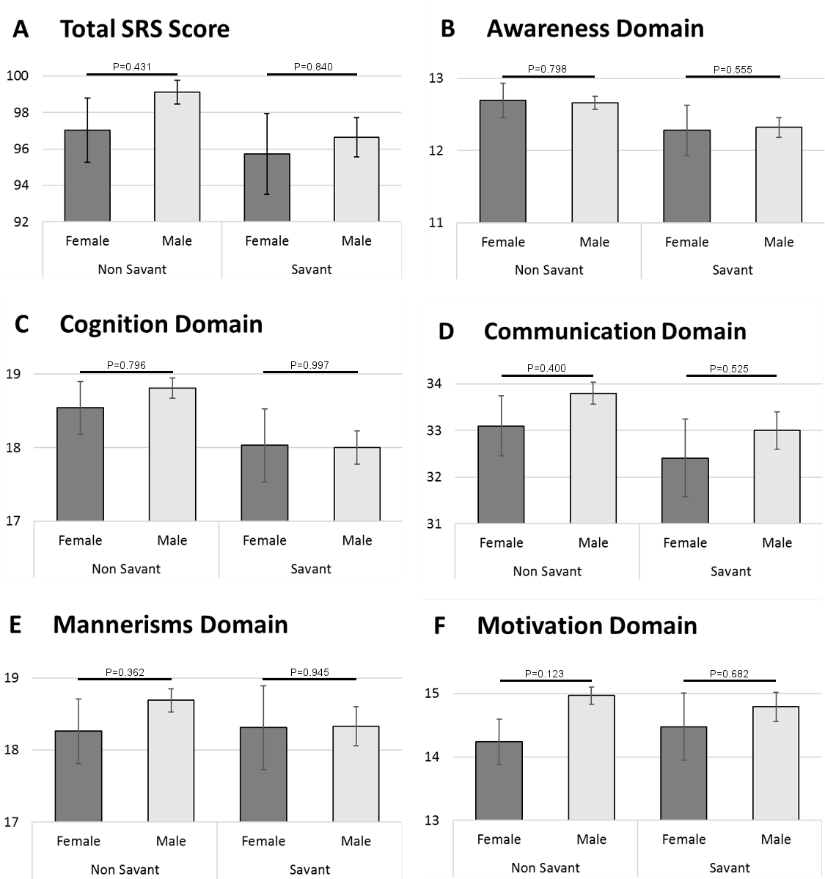
**

**Figure S2**

Gender differences in proband raw SRS scores. Gender-specific mean ± SE of SRS scores are displayed for the non-savant (Dark Gray bars), and savant (Light Gray bars) groups. (A) Total SRS scores; (B) awareness domain scores; (C) cognition domain scores; (D) communication domain scores; (E) mannerism domain scores; (F) motivation domain scores.
